# Comparing different ways of calculating sample size for two independent means: A worked example

**DOI:** 10.1016/j.conctc.2018.100309

**Published:** 2018-11-29

**Authors:** Lei Clifton, Jacqueline Birks, David A. Clifton

**Affiliations:** a Centre for Statistics in Medicine (CSM), NDORMS, University of Oxford, United Kingdom; b Institute of Biomedical Engineering (IBME), Department of Engineering Science, University of Oxford, United Kingdom

**Keywords:** Sample size, Independent, Means, Standard error, Standard deviation, RCT, Variance, Change score, Baseline, Correlation, Arm, Covariate, Outcome measure, Post-intervention

## Abstract

We discuss different methods of sample size calculation for two independent means, aiming to provide insight into the calculation of sample size at the design stage of a parallel two-arm randomised controlled trial (RCT). We compare different methods for sample size calculation, using published results from a previous RCT. We use variances and correlation coefficients to compare sample sizes using different methods, including

1. The choice of the primary outcome measure: post-intervention score vs. change from baseline score.

2. The choice of statistical methods: *t*-test without using correlation coefficients vs. analysis of covariance (ANCOVA).

We show that the required sample size will depend on whether the outcome measure is the post-intervention score, or the change from baseline score, with or without baseline score included as a covariate. We show that certain assumptions have to be met when using simplified sample size equations, and discuss their implications in sample size calculation when planning an RCT. We strongly recommend publishing the crucial result “mean change (SE, standard error)” in a study paper, because it allows (i) the calculation of the variance of the change score in each arm, and (ii) to pool the variances from both arms. It also enables us to calculate the correlation coefficient in each arm. This subsequently allows us to calculate sample size using change score as the outcome measure. We use simulation to demonstrate how sample sizes by different methods are influenced by the strength of the correlation.

## 1 Background

Sample size calculations for a parallel two-arm trial with a continuous outcome measure can be undertaken based on (i) a pre-specified difference between arms at the post-intervention endpoint and (ii) an estimate of the standard deviation (SD) of the outcome measure. If the outcome variable is also measured at baseline, an alternative outcome measure is *change from baseline* instead of the post-intervention measure. Use of this alternative outcome measure would result in a different power calculation from that obtained using the post-intervention as the outcome measure. It is possible to carry out a power calculation based on analysis of covariance (ANCOVA) where the baseline measure is included as a covariate in the analysis.

Sample size calculations typically use published results from trials similar to those under consideration. We use results from a published paper for the MOSAIC trial [1] to compare different methods for sample size calculation. We examine the assumptions made by each method for calculating sample size, and discuss the implications of these assumptions when calculating the required sample size for a new RCT. We aim to provide insight into sample size calculations at the design stage of an RCT.

We introduce the notion of change scores, and show how to derive variances of these change scores along with related correlation coefficients in Section 3, using published results. We then calculate and compare sample sizes using different methods in Section 4. A description of the simulation of different strengths of the correlation is presented in Section 5, with the aim of investigating its influence on the calculation of sample sizes using different methods. Section 6 discusses simplified sample size equations when certain assumptions are met. Finally, we consider implications in sample size calculation when planning an RCT in Section 7.

## 2 Method

### 2.1 Published results of the MOSAIC trial

The MOSAIC trial is an RCT using continuous positive airway pressure (CPAP) for symptomatic obstructive sleep apnoea. The trial randomised 391 patients between two treatment arms (CPAP vs. standard care). It has two primary outcomes at 6 months: change in Epworth Sleepiness Score (ESS), and change in predicted 5-year mortality using a cardiovascular risk score. The authors also reported the energy/vitality score (referred to as the “energy score” hereafter) of the 36-item short-form questionnaire (SF-36). The change in SF-36 energy score at 6 months is a secondary outcome of the MOSAIC trial, and an investigator might conduct another RCT using it as the primary outcome. The online supplement of the MOSAIC paper [1] states that all data were analysed using multiple variable regression models adjusting for the minimisation variables and baseline value of the variable being analysed.

Table 1 shows data concerning the SF-36 energy score, taken from Table 4 in the MOSAIC paper [1]. The outcome measure is energy score in the SF-36 questionnaire, measured at baseline and at 6 months post-intervention. An increase in the energy score indicates an improvement in health status. The table shows that the adjusted treatment effect (6.6) is the same as the unadjusted treatment effect (10.8–4.2 = 6.6). The baseline mean scores are similar in both arms, being 49.7 and 49.8, respectively.

**Table 1:** SF-36 energy score at baseline and 6-month post-intervention, reproduced using results from the MOSAIC trial.

Energy	Control arm (N = 168)	CPAP arm (N = 171)
Baseline mean score (SD)	49.7 (23.7)	49.8 (22.4)
6-month mean score (SD)	53.9 (22.5)	60.6 (20.9)
Mean change (SE)	+4.2 (1.4)	+10.8 (1.3)
Adjusted treatment effect (95% CI)	+6.6 (+3.1 to +10.1)	
p value	p < 0.0001	

CPAP, continuous positive airway pressure; SF-36, 36-item Short-Form health survey; SD, standard deviation; SE, standard error; CI, confidence interval; N, number of participants.

In the following sections, we show how to derive the variances of the change scores and correlation coefficients between baseline and 6 month measurements for both arms, using the results reported in Table 1 including “Mean change (SE)“.

### 2.2 Deriving the sample variance of the change score $\;\left(Y_{1}-Y_{0}\right)$

We use generic notation in this paper, noting that the proposed method is applicable to arbitrary continuous outcome measures. Suppose the primary continuous outcome measure is Y, with $Y_{0}$ and $Y_{1}$ denoting Y at baseline and post-intervention, respectively. For simplicity, we will call $Y_{0}$ the “baseline score”, $Y_{1}$ the “post score”, and $\left(Y_{1}-Y_{0}\right)$ the “change score”.

Let $s_{Y_{0}}^{2}$ denote the sample variance of baseline score $Y_{0}$, $s_{Y_{1}}^{2}$ denote the sample variance of post score $Y_{1}$, $s_{\left(Y_{1}-Y_{0}\right)}^{2}$ denote the sample variance of the change score $\left(Y_{1}-Y_{0}\right)$. Let $s_{Y_{0}}$, $s_{Y_{1}}$, and $s_{\left(Y_{1}-Y_{0}\right)}$ denote their corresponding standard deviations (SD). We show how to derive $s_{\left(Y_{1}-Y_{0}\right)}^{2}$ in each arm, for the purpose of calculating sample size.

Let $se_{\left(Y_{1}-Y_{0}\right)}$ denote the standard error (SE) of $\left(Y_{1}-Y_{0}\right)$, and N denote the number of participants; $se_{\left(Y_{1}-Y_{0}\right)}$ can then be expressed

$$se_{\left(Y_{1}-Y_{0}\right)}=\sqrt{s_{\left(Y_{1}-Y_{0}\right)}^{2}/N}$$

$$∴s_{\left(Y_{1}-Y_{0}\right)}^{2}=se_{\left(Y_{1}-Y_{0}\right)}^{2}\cdot N$$

The SEs reported in Table 1 (which are 1.4 and 1.3 in the control and intervention arms, respectively) are those results that allow us to derive $s_{\left(Y_{1}-Y_{0}\right)}^{2}$ using the relationship above.

For the control arm, using the formulation above, we have $s_{\left(Y_{1}-Y_{0}\right)}^{2}=se_{\left(Y_{1}-Y_{0}\right)}^{2}\cdot N=1.4^{2}\cdot 168=$ $18.15^{2}$. For the intervention arm, we have $s_{\left(Y_{1}-Y_{0}\right)}^{2}=se_{\left(Y_{1}-Y_{0}\right)}^{2}\cdot N=1.3^{2}\cdot 171=17.00^{2}$. These derived values $s_{\left(Y_{1}-Y_{0}\right)}^{2}$ of $\;18.15^{2}$ and $17.00^{2}$ are different in the two treatment arms; therefore, we will need to use their pooled variance for the calculation of sample size. Using the equation shown in the Appendix, the pooled sample variance of $\left(Y_{1}-Y_{0}\right)$ is

$$s_{p,\left(Y_{1}-Y_{0}\right)}^{2}=\frac{\left(168-1\right)18.15^{2}+\left(171-1\right)17.00^{2}}{168+171-2}=309.03=17.58^{2}$$

The calculation of $s_{\left(Y_{1}-Y_{0}\right)}^{2}$ above requires the knowledge of “mean change (SE)” reported in Table 1. The presence of r is implicitly acknowledged, and we will use $s_{\left(Y_{1}-Y_{0}\right)}^{2}$ to derive the value of r in the next section.

### 2.3 Deriving correlation coefficient r between $Y_{0}$ and $Y_{1}$

This section shows how to use the variance sum law to derive the correlation coefficient r between $Y_{0}$ and $Y_{1}$. The variance sum law states

(1) $$s_{\left(Y_{1}-Y_{0}\right)}^{2}=s_{Y_{0}}^{2}+s_{Y_{1}}^{2}-2\;r\;s_{Y_{0\;}}s_{Y_{1}}$$

Let $r_{c}$ and $r_{t}$ denote r in the control and intervention arms, respectively. Substituting $s_{Y_{0}}^{2}$, $s_{Y_{1}}^{2}$, and our derived $s_{\left(Y_{1}-Y_{0}\right)}^{2}$ into the variance sum law above, we have $r_{c}=0.6925$ and $r_{t}=0.6937$. Table 2 summarise the sample variances and correlation coefficients for the exemplar study. Here we have explicitly calculated the value of r using $s_{\left(Y_{1}-Y_{0}\right)}^{2}$ derived in the previous section.

**Table 2:** Summary of sample variances.

Energy score	Control arm (N = 168)	CPAP arm (N = 171)	Pooled
Variance of baseline score, $s_{Y_{0}}^{2}$	$23.7^{2}$	$22.4^{2}$	$23.1^{2}$
Variance of post score, $s_{Y_{1}}^{2}$	$22.5^{2}$	$20.9^{2}$	$21.7^{2}$
Variance of change score, $s_{\left(Y_{1}-Y_{0}\right)}^{2}$	$18.15^{2}$	$17.00^{2}$	$17.58^{2}$
Correlation between baseline and post scores	0.6925	0.6937	–

The derived $r_{c}$ and $r_{t}$ are very similar, being approximately equal to 0.7; therefore, we will use $r=r_{c}=r_{t}=0.7$ for the sample size calculation in the following sections. We note that if $r_{c}\neq r_{t}$, the sample size method via ANCOVA in this paper will not be valid; in this example, the values of $r_{c}$ and $r_{t}$ are very close, granting the validity of using ANCOVA for sample size calculation. We will discuss the implication of different values for $r_{c}$ and $r_{t}$ in later Sections.

## 3 Comparing different sample size calculations

The calculation of sample size will depend on whether the outcome measure is to be the post score or the change score, without and with baseline included as a covariate.

### 3.1 Sample size: *t*-test on post score $\;Y_{1}$

Using $Y_{1}$ as the outcome measure in our example, the pooled variance of $\;Y_{1}$ is (see Appendix)

$$s_{p,Y_{1}}^{2}=\frac{\left(168-1\right)22.5^{2}+\left(171-1\right)20.9^{2}}{168+171-2}=471.22=21.7^{2}$$

For a two-sided significance level α at power 1−β, with pooled variance of $s_{p}^{2}$, the required number of patients per arm is approximately [2].

(2) $$N=\;\frac{2\;{\left(z_{1-\alpha /2}+z_{1-\beta }\right)}^{2}s_{p}^{2}}{\delta^{2}}$$

where $\delta =\mu_{2}-\mu_{1}$ is the target mean difference between the two treatment arms, and where $z_{1-\alpha /2}$ and $z_{1-\beta }$ are the ordinates for the standard normal distribution, z∼N(0,1). If assuming equal variance $\sigma^{2}$, simply substitute $s_{p}^{2}$ for $\sigma^{2}$ in Equation (2).

In the exemplar considered by this paper, we use two-sided significance level α=0.05, and power 1−β=0.8, corresponding to $z_{1-\alpha /2}=z_{0.975}=1.96$, and $z_{1-\beta }=z_{0.8}=0.842$, respectively.

In our example, the target mean difference is set to be the reported treatment effect in Table 1, δ=6.6. The variances of the two arms are different, and we have calculated the pooled variance $s_{p}^{2}=\;$ $21.7^{2}$. The required number of patients per arm is approximately

$$N=\;\frac{2\;{\left(1.96+0.842\right)}^{2}\;21.7^{2}}{6.6^{2}}=169.7\approx 170$$

In the trial design stage, the characteristics of the planned RCT will inevitably differ from those of a previously-published trial, and it is therefore desirable to calculate sample sizes over a range of variances. For example, assuming equal variance using $\sigma^{2}=s_{X}^{2}=22.5^{2}$ and $\sigma^{2}\;=s_{Y}^{2}=20.9^{2}$ in Equation (2), the resulting sample sizes are N=183 and N=158, respectively. The pooled variance produces a modest sample size N=170. In practice, one may choose to calculate N using the most conservative (i.e., the greatest) value of variances when designing a new RCT.

### 3.2 Sample size: *t*-test on change score $\left(Y_{1}-Y_{0}\right)$

When using change score $(Y_{1}-Y_{0}$) as the outcome measure, we can still use Equation (2) to calculate N, using the pooled variance of $(Y_{1}-Y_{0}$), $s_{p,\left(Y_{1}-Y_{0}\right)}^{2}$. We have derived $s_{p,\left(Y_{1}-Y_{0}\right)}^{2}=17.58^{2}$ in the previous section; substituting the latter into Equation (2) gives

$$N=\;\frac{2\;{\left(1.96+0.842\right)}^{2}\;17.58^{2}}{6.6^{2}}=111.4\approx 112$$

For comparison, if we assume equal variance using $\sigma^{2}=s_{X}^{2}=18.15^{2}$ and $\sigma^{2}\;=s_{Y}^{2}=17.00^{2}$ in Equation (2), the resulting sample sizes are N=119 and N=105, respectively. The pooled variance produces a modest sample size N=112. We have used this pooled variance $s_{p,\left(Y_{1}-Y_{0}\right)}^{2}=17.58^{2}$ in the sample size calculation shown in Table 3.

**Table 3:** Comparing sample sizes using different outcome measures and statistical methods.

Outcome	N in each arm	
	ANCOVA	*t*-test
$Y_{1}$	87 (85)	170 (171)
($Y_{1}-Y_{0}$)	–	112 (113)

N, number of patients in each arm. N calculated by equation are shown together with N produced by PASS software: N by equation (N by PASS).

We strongly recommend publishing resulting “mean change (SE)” in a study paper, because it allows the calculation of $s_{\left(Y_{1}-Y_{0}\right)}^{2}$ in each arm, and to pool the variances from both arms. We note here that deriving $s_{\left(Y_{1}-Y_{0}\right)}^{2}$ does not required the knowledge of the correlation coefficient r between $Y_{0}$ and $Y_{1}$, as long as the SE of $\left(Y_{1}-Y_{0}\right)$ is reported. As shown in previous sections, the derived $s_{\left(Y_{1}-Y_{0}\right)}^{2}$ enables us to calculate r in each arm. This subsequently allows us to calculate sample size using the change score $(Y_{1}-Y_{0}$) as the outcome measure. We will use the derived r to calculate N via ANCOVA in the next section.

### 3.3 Sample size: assumptions of ANCOVA on $Y_{1}$ adjusting for $Y_{0}$

When using $Y_{1}$ as the outcome while adjusting for $Y_{0}$, the sample size N can be calculated via ANCOVA. Let $\tau^{2}$ and $\sigma^{2}$ be the variances of $Y_{0}$ and $Y_{1}$, respectively. Let $\left(Y_{0,\;i,j},\;Y_{1,i,j}\right)$ be the paired data of $Y_{0}$ and $Y_{1}$, where i=1,2 represents the two treatment arms, and where j=1,…,N represents each of the N patients.

If we assume $\left(Y_{0,\;i,j},\;Y_{1,i,j}\right)$ follow a bivariate normal distribution, then the distribution of $\left(Y_{1}|Y_{0}\right)$, which is $Y_{1,i,j}$ conditioned on $Y_{0,i,j}$, is a univariate normal distribution with a variance of $\sigma^{2}\left(1-r^{2}\right)$, as shown in the Appendix. We note that $\tau^{2}$, the variance of the baseline score $Y_{0}$, does not appear in the conditional variance of $\left(Y_{1}|Y_{0}\right)$. This relationship indicates a variance deflation factor $\left(1-r^{2}\right)$ that can be used for sample size calculation.

However, this variance deflation factor is only true under the assumption of a bivariate normal distribution of $\left(Y_{0,\;i,j},\;Y_{1,i,j}\right)$. As stated above, this means that the marginal distribution of $Y_{0}$ is normal, and that the marginal distribution of $Y_{1}$ is also normal, hence the usual assumed normality for a *t*-test is met. However, the marginal normal distributions of $Y_{1}$ and $Y_{0}$ do not guarantee the bivariate normal distribution of $\left(Y_{0,\;i,j},\;Y_{1,i,j}\right)$. Therefore, the assumption of a bivariate normal distribution of $\left(Y_{0,\;i,j},\;Y_{1,i,j}\right)$ is a stronger assumption than the assumption in a *t*-test for sample size, and can be violated in practice. It is necessary to examine assumption of a bivariate normal distribution of $\left(Y_{0,\;i,j},\;Y_{1,i,j}\right)$ before applying the variance deflation factor in the sample size calculation.

It is straightforward to visualise $\left(Y_{0,\;i,j},\;Y_{1,i,j}\right)$ by plotting the data in a two-dimensional space, with treatment arm on the horizontal axis, and $\left(Y_{0,\;i,j},\;Y_{1,i,j}\right)$ on the vertical axis. This visualisation will immediately reveal whether the assumption of a bivariate normal distribution is violated. It is possible that data will form two clusters corresponding to the control and intervention arms, respectively, which therefore violates the assumption. Borm, Fransen et al. [3], used this relationship for sample size calculation via ANCOVA, but the authors did not explicitly discuss its assumption.

There are several other assumptions one must make before applying the variance deflation factor $\left(1-r^{2}\right)$ . In this paper, we give mathematical details in the Appendix and explicitly examine all the assumptions, summarised below:

1. All pairs $\left(Y_{0},Y_{1}\right)$, including all patients in both arms, follow a bivariate normal distribution. We recommend visualising the data to examine whether this assumption is violated, as discussed above.
2. The values of the correlation coefficient r between $Y_{0}$ and $Y_{1}$ are the same in both arms. This means that there exists no interaction between baseline score and the treatment arm. This assumption is adequately met in our example, where r≈0.7 in both arms of the trial.
3. The variances of $Y_{1}$ , denoted $\sigma^{2}$, are the same in both arms. We note that the variance of $Y_{0}$, denoted $\tau^{2}$, does not affect the variance deflation factor, hence it does not have to take the same value in both arms. This assumption is *mildly* violated in our example, because Table 2 shows that the pooled $s_{Y_{0}}^{2}$ and $s_{Y_{1}}^{2}$ are quite similar, being $23.1^{2}$ and $21.7^{2}$, respectively. The resulting sample size by ANCOVA shown in Table 3 should still be a reasonable estimate, due to these similar values of the pooled $s_{Y_{0}}^{2}$ and $s_{Y_{1}}^{2}$.

If all of the above assumptions hold, then the conditional variance of $\left(Y_{1}|Y_{0}\right)$ is $\sigma^{2}\left(1-r^{2}\right)$, indicating a variance deflation factor of $\left(1-r^{2}\right)$. Let N be the sample size (i.e., the number of patients in each arm) by a *t*-test on $Y_{1}$, then the sample size by an ANCOVA on $Y_{1}$ adjusting for $Y_{0}$ is

(3) $$N_{A}=\left(1-r^{2}\right)N$$

while achieving the same power as a *t*-test on $Y_{1}$. Since $\left(1-r^{2}\right)N\leq N$, ANCOVA always produces a smaller sample size than a *t*-test, illustrated in the first row of Table 3.

In our example, the variance of $Y_{1}$ in the control and intervention arms is different ($22.5^{2}$ and $20.9^{2}$, respectively), hence it does not meet the assumption of equal variance above (#3).

### 3.4 Comparing sample sizes using different methods

This section summarises and compares different methods for sample size calculation. We discuss the following two factors:

1. The choice of the primary outcome measure: post score $Y_{1}$ vs. change score $(Y_{1}-Y_{0}$).
2. The choice of statistical methods: *t*-test without using r vs. ANCOVA.

In all sample size calculations in this paper (including those for which the results are shown in Table 3), we have used the target mean difference δ=6.6, two-sided α=0.05, allocation ratio = 1, achieving 80% power. All sample sizes are produced using the corresponding pooled variance derived in this paper. We used the PASS 15 system (NCSS, LLC) to validate our sample size calculation by equations, shown as “(N by PASS)” in Table 3, and where “N by equation” refers to our derived N in previous sections. The algorithm implemented by the PASS software uses Borm, Fransen et al. [3], in its reference for sample size via ANCOVA, and its results (“N by PASS”) are similar to the “N by equation”.

The efficiency (i.e. smaller N while maintaining the same statistical power) gained in ANCOVA by using r comes from making strong assumptions. We have used Equation (3) from Section 4.3 (i.e., sample size via ANCOVA) in Table 3, but we note that its assumptions are not fully met in individual arms, and therefore one should not directly use the variance of individual arms for the sample size calculation in ANCOVA. In this instance, our approach is to use the pooled variance of both arms in the sample size equation via ANCOVA. Acknowledging its limitation in practice, one can produce sample sizes using a range of variances to gain a better sense of the required sample size.

In Table 3, we have used r=0.7 for sample size via ANCOVA, as stated previously. In both the “*t*-test” and “ANCOVA” methods, we have used the pooled variance $s_{p}^{2}=\;$ $21.7^{2}$ for the *t*-test on $Y_{1}$, and $s_{p,\left(Y_{1}-Y_{0}\right)}^{2}=17.58^{2}$ for the *t*-test on ($Y_{1}-Y_{0}$).

In the example corresponding to the results shown in Table 3, ANCOVA produces the smallest sample size, while use of a *t*-test on $Y_{1}$ produces the largest. Calculating sample size via a *t*-test for outcome $Y_{1}$ does not consider the correlation r between $Y_{0}$ and $Y_{1}$, hence will always yield a sample size larger than that obtained when using an ANCOVA (which involves the use of the value of r). However, N via a *t*-test for outcome ($Y_{1}-Y_{0}$) is not always larger than N via ANCOVA, depending on the strength of the correlation r and meeting the assumptions presented earlier.

## 4 Simulated sample sizes at different values of r

We here simulate different values of r, and then compare the sample sizes calculated using different methods. The pooled variances $s_{Y_{0}}^{2}$, $s_{Y_{1}}^{2}$, and $s_{\left(Y_{1}-Y_{0}\right)}^{2}$ are used in all simulations in this section. The variance sum law shown in Equation (1) indicates that we have the following two options for simulation when varying the value of r:

**Option 1:** Keeping the variance of the change score (i.e., $s_{\left(Y_{1}-Y_{0}\right)}^{2}$) fixed at the derived value of $17.58^{2}$. The implication is that $s_{Y_{0}}^{2}$ and $s_{Y_{1}}^{2}$ are allowed to vary according to r.
**Option 2:** Allowing the variance of the change score to vary with r, while keeping $s_{Y_{0}}^{2}$ and $s_{Y_{1}}^{2}$ fixed at the derived values, $23.1^{2}$ and $21.7^{2}$, respectively.

**Fig. 1:**
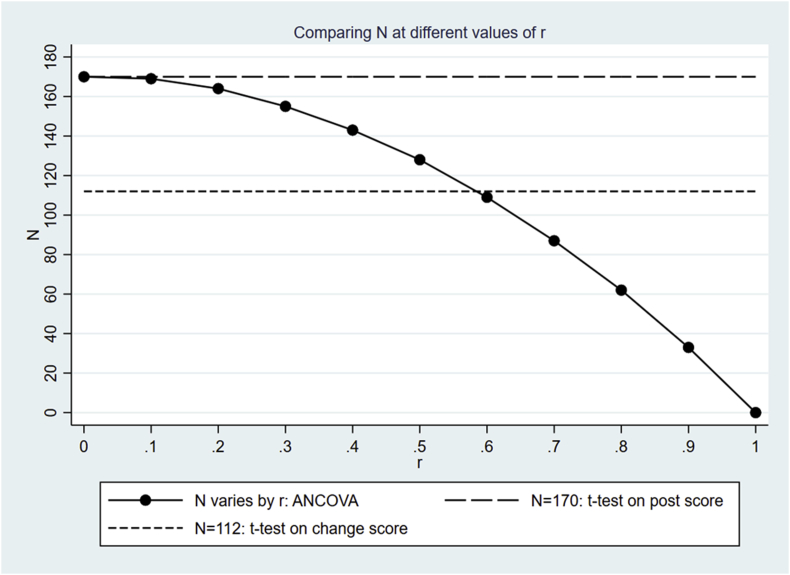
Comparing values of sample size N produced using different methods at different values of r, using the same parameter values as are shown in Table 3. The values of $s_{\left(Y_{1}-Y_{0}\right)}^{2}$ remain fixed for all values of r, resulting in a constant value of N=112 via a *t*-test for outcome ($Y_{1}-Y_{0}$), shown by the short-dashed line. Fig. 1 is intended to be compared with Fig. 2, where the values of $s_{\left(Y_{1}-Y_{0}\right)}^{2}$ are allowed to vary according to the values of r.

**Fig. 2:**
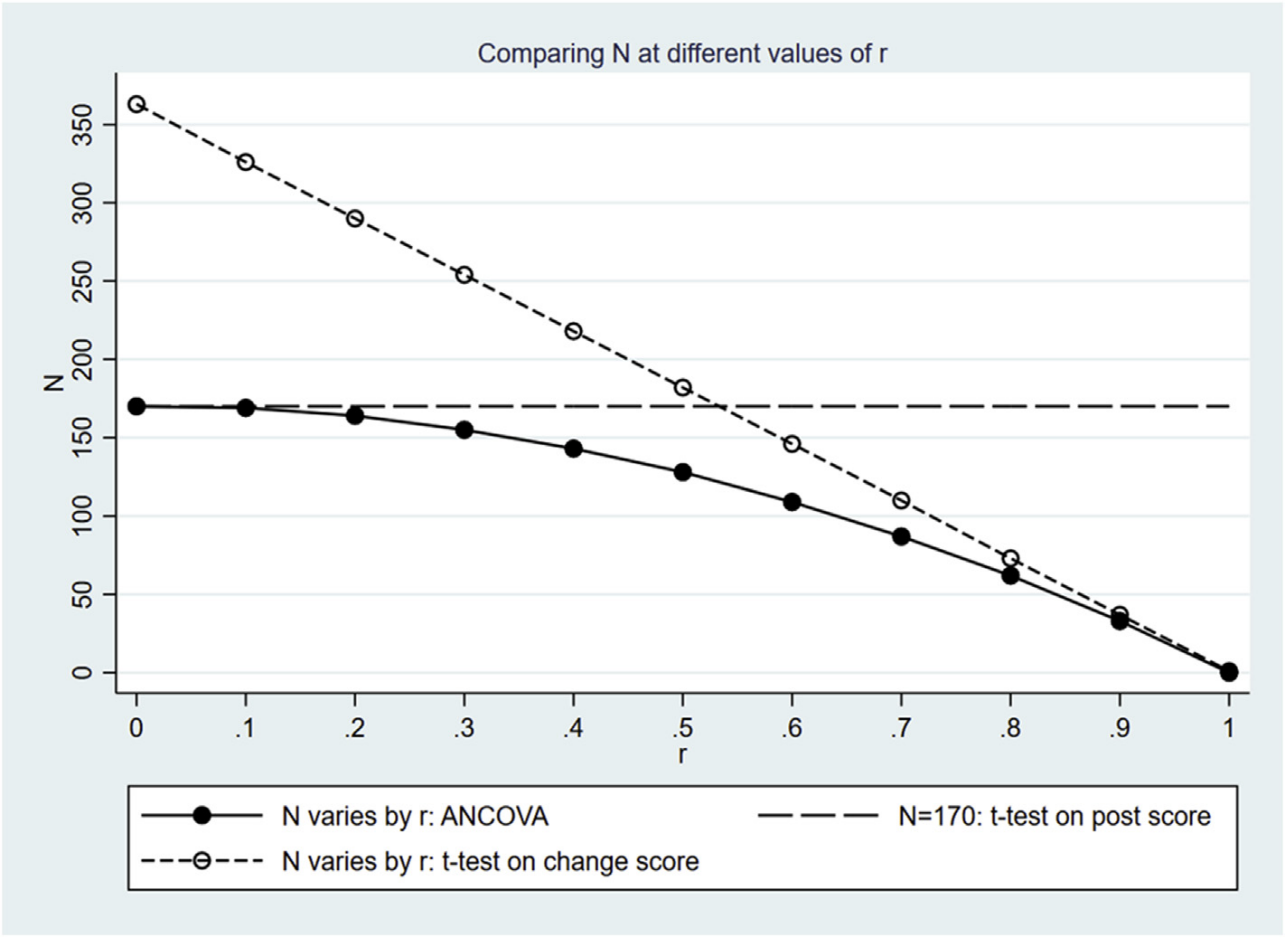
Similar to Fig. 1 above, except that the values of $s_{\left(Y_{1}-Y_{0}\right)}^{2}$ are allowed to vary according to the values of r. Note that the range of the y-axis here is different from that in Fig. 1.

We show the simulated sample sizes of these two options above in the following sections. The simulated results using both options are shown in Table 4 below, and are plotted in Fig. 1 and Fig. 2. The same parameter values as presented in Table 3 are used for simulation throughout this section.

**Table 4:** Simulated sample sizes at different values of r. “N by ANCOVA” produced by option 1 (plotted in Fig. 1) are the same as those produced by option 2 (plotted in Fig. 2). “N by *t*-test on post score” remains at a constant value of 170 throughout. In contrast, “N by *t*-test on change score” by option 1 and 2 are different, and are plotted in Figs. 1 and 2, respectively.

Correlation r	0	0.1	0.2	0.3	0.4	0.5	0.6	0.7	0.8	0.9	1.0
N by ANCOVA	170	169	164	155	143	128	109	87	62	33	0
N by *t*-test on post score	170	170	170	170	170	170	170	170	170	170	170
N by *t*-test on change score (Fig. 1)	112	112	112	112	112	112	112	112	112	112	112
N by *t*-test on change score (Fig. 2)	363	326	290	254	218	182	146	110	73	37	0

### 4.1 Option 1: keeping the variance of the change score fixed

Fig. 1 compares sample sizes obtained using option 1 above using different methods at different values of r. Sample size N via a *t*-test for outcome $Y_{1}$ is shown in long-dashed line, calculated using the equation in Section 4.1. Sample size N via a *t*-test for outcome ($Y_{1}-Y_{0}$) is shown in short-dashed line, calculated using the equation in Section 4.2. The value of N produced by both above options is not influenced by the correlation r, hence remains the same at different values of r. In contrast, the values of N for outcome $Y_{1}$ via ANCOVA, produced by Equation (3) in Section 4.3, heavily depend on the value of r; the larger the value of the correlation r, the smaller the resulting value of N.

The results shown in Table 3 correspond to values of N at r=0.7, where the value of N obtained via ANCOVA is smaller than the value of N obtained via a *t*-test on the outcome ($Y_{1}-Y_{0}$). However, N by ANCOVA becomes larger than N by a *t*-test ($Y_{1}-Y_{0}$) once r decreases to values below 0.6, as shown in Fig. 1. The value of N obtained via a *t*-test on the outcome $Y_{1}$ remains the largest among the three methods at all values of r.

### 4.2 Option 2: varying the variance of the change score according to r

Alternatively, we can allow the values of $s_{\left(Y_{1}-Y_{0}\right)}^{2}$ to vary according to r, while keeping the values of $s_{Y_{0}}^{2}$ and $s_{Y_{1}}^{2}$ fixed in Equation (1). Fig. 2 shows the resulting sample sizes obtained by the three different methods, to be compared with Fig. 1. In Fig. 2, the resulting N via ANCOVA remain the same as those shown in Fig. 1, but N via a *t*-test for outcome ($Y_{1}-Y_{0}$) are different from those in Fig. 1 due to varying $s_{\left(Y_{1}-Y_{0}\right)}^{2}$ by the values of r.

Fig. 2 also provides a convenient way of assessing the assumption of equal variance required in Equation (4). If the assumption that $Y_{0}$ and $Y_{1}$ have the same variance is met, the long-dashed line in Fig. 2 (representing the value of N obtained via a *t*-test on $Y_{1}$) and the short-dashed line (representing the value of N obtained via a *t*-test on $Y_{1}-Y_{0}$) will cross at r=0.5. These two lines cross at r=0.53 in Fig. 2, indicating this assumption is only *mildly* violated.

## 5 Simplified sample size equations under assumptions

### 5.1 The variance sum law when assuming equal variance

Assuming $Y_{0}$ and $Y_{1}$ have the same variance $\sigma^{2}$, the variance sum law (Equation (1)) can be simplified to

(4) $$s_{\left(Y_{1}-Y_{0}\right)}^{2}=\sigma^{2}+\sigma^{2}-2\;r\;\sigma^{2}=2\;\left(1-r\right)\;\sigma^{2}$$

This means that when $(Y_{1}-Y_{0}$) is the outcome measure, its variance deflation factor is 2(1−r), assuming that $Y_{0}$ and $Y_{1}$ have an equal variance $\sigma^{2}$. This variance deflation factor gives us a simplified Equation (4) for sample size. Let N be the sample size (i.e., the number of patients in each arm) obtained by a *t*-test on $Y_{1}$; then a *t*-test on $(Y_{1}-Y_{0}$) will require 2(1−r)N patients to achieve the same power, assuming equal variance of $Y_{0}$ and $Y_{1}$.

Since 2(1−r)N>N, if r>0.5, and vice versa if r<0.5, then Equation (4) also shows that calculating sample size using a *t*-test on $(Y_{1}-Y_{0}$) will require fewer patients than would be obtained were a *t*-test on $Y_{1}$ used, if r>0.5 and vice versa if r<0.5. The two methods yield the same number of patients if r=0.5. We emphasise that this relationship only strictly applies when $Y_{0}$ and $Y_{1}$ have equal variance $\;\sigma^{2}$. In practice, if $s_{Y_{0}}^{2}$ and $s_{Y_{1}}^{2}$ are sufficiently similar in value, Equation (4) can still give a reasonable estimate of $s_{\left(Y_{1}-Y_{0}\right)}^{2}$, and hence give a reasonable estimate of sample size. This is further illustrated by Fig. 2, where the long-dashed and short-dashed lines cross at r=0.53, a close value to 0.5, indicating a *mild* violation of the assumption on equal variance.

In our example, Table 2 shows that $Y_{0}$ and $Y_{1}$ do not have equal variance, hence the above formula is not directly applicable. However, Table 2 also shows that the values of pooled $s_{Y_{0}}^{2}$ and $s_{Y_{1}}^{2}$ are quite similar, being $23.1^{2}$ and $21.7^{2}$, respectively. In practice, one can use Equation (4) to calculate $s_{\left(Y_{1}-Y_{0}\right)}^{2}$ assuming $s_{Y_{0}}^{2}$ and $s_{Y_{1}}^{2}$ are the same, to be compared with the derived $s_{\left(Y_{1}-Y_{0}\right)}^{2}=17.58^{2}\;$ using actual results. It turns out that if $s_{Y_{0}}^{2}=s_{Y_{1}}^{2}=22.0^{2}$, Equation (4) will yield $s_{\left(Y_{1}-Y_{0}\right)}^{2}=17.04^{2}$, which is quite similar to our derived $s_{\left(Y_{1}-Y_{0}\right)}^{2}=17.58^{2}\;$.

### 5.2 Sample sizes when all assumptions are met

Let N be the sample size by a *t*-test on $Y_{1}$. If all assumptions discussed in Section 4.3 and Section 5.1 are met, calculating sample size via ANCOVA on $Y_{1}$ while adjusting for $Y_{0}$ will require $\left(1-r^{2}\right)N$ patients in total, whereas using a *t*-test on $(Y_{1}-Y_{0}$) will require 2(1−r)N patients.

Using ${\left(r-1\right)}^{2}\geq 0$, we have

(5) $$\left(1-r^{2}\right)N\leq 2\left(1-r\right)N$$

where equality occurs at r=1. The left hand and right hand sides of Equation (5) correspond to the sample size obtained via ANCOVA on $Y_{1}$ while adjusting for $Y_{0}$ and via a *t*-test on $\;(Y_{1}-Y_{0}$), respectively. In practice, we always have r<1; therefore ANCOVA on $Y_{1}$ adjusting for $Y_{0}$ always yields a smaller sample size than would be obtained using a *t*-test on $(Y_{1}-Y_{0}$), if all assumptions hold. Fig. 2 in Section 5.2 also illustrates Equation (5), where the short-dashed line showing N by *t*-test on $(Y_{1}-Y_{0}$) is always above the solid line showing N by ANCOVA on $Y_{1}$ adjusting for $Y_{0}$, except at r=1.

## 6 Discussion

### 6.1 The implications of correlation coefficient r

When designing a new RCT, one needs to consider whether the duration of the planned trial will differ from that of previous trials. The correlation between $Y_{0}$ and $Y_{1}$ is likely to decrease (i.e., a smaller r) for an increased trial period, and vice versa.

In the example used in this paper, the derived correlation coefficient r is similar in both treatment arms, being approximately 0.7. If the correlation between $Y_{0}$ and $Y_{1}$ in the two treatment arms is different, one will need to consider the interaction between the treatment arm and baseline measure.

### 6.2 If “mean change (SE)” is not reported

If “mean change (SE)” is not reported for a study, we can calculate a range of potential variances of $(Y_{1}-Y_{0}$) by setting a plausible range of values of r, using the variance sum law, as shown in Section 3.3. The simulation method shown in Section 5 can be used to compare sample sizes obtained using different methods at different values of r, providing a sense of the required sample size in the trial design stage.

### 6.3 Future work

In this paper we have used change score $(Y_{1}-Y_{0}$) as a choice of outcome measure without questioning its validity. In fact, one should be cautious of using change score as the outcome measure, due to the well-known statistical phenomenon of “regression to the mean”. This will be investigated in a future paper.

## Declarations

### Ethics approval and consent to participate

N/A. Not required.

### Consent for publication

Yes.

### Availability of data and material

N/A. Not required.

### Competing interests

None.

### Funding

N/A.

### Authors' contributions

LC conceived the research idea, and led the writing of the paper. JB and DC also contributed to writing the paper.

## Appendix

### Pooled sample variance

Let $s_{X}^{2}$ and $s_{Y}^{2}$ denote the sample variance of the control and intervention arms, respectively. Let n and m denote the number of patients in the control and intervention arms, respectively. The pooled sample variance is [4] p.349

$$s_{p}^{2}=\frac{\left(n-1\right)s_{X}^{2}+\left(m-1\right)s_{Y}^{2}}{m+n-2}$$

The value of $s_{p}$ is between $s_{X}$ and $s_{Y}$. In the special case of equal variance $s_{X}^{2}=s_{Y}^{2}=\;\sigma^{2}$, the pooled variance $s_{p}^{2}$ is simplified to the shared variance $\sigma^{2}$.

### Bivariate normal distribution

Here we use the more general symbols Y to denote the post-score, and X to denote the baseline score. Let r be the correlation coefficient between X and Y. The bivariate normal density is given by the expression [4] p.75

$$f\left(x,\;y\right)=\frac{1}{2\pi \sigma_{X}\sigma_{Y}\sqrt{\left(1-r^{2}\right)}}\exp\left(-\frac{1}{2\left(1-r^{2}\right)}\left[\frac{{\left(x-\mu_{X}\right)}^{2}}{\sigma_{X}^{2}}+\frac{{\left(y-\mu_{Y}\right)}^{2}}{\sigma_{Y}^{2}}-\frac{2r\left(x-\mu_{X}\right)\left(y-\mu_{Y}\right)}{\sigma_{X}\sigma_{Y}}\right]\right)$$

The marginal distribution of X and Y are $N\left(\mu_{X},\sigma_{X}^{2}\right)$ and $N\left(\mu_{Y},\sigma_{Y}^{2}\right)$, respectively.

### Conditional distribution of a bivariate normal distribution

The conditional density of Y given X is the ratio of the bivariate normal density to a univariate normal density [4] p.83. This ratio simplifies to

$$f_{Y|X}\left(y|x\right)=\frac{1}{\sigma_{Y}\sqrt{2\pi \left(1-r^{2}\right)}}\exp\left(-\frac{1}{2}\frac{{\left[y-\mu_{Y}-r\frac{\sigma_{Y}}{\sigma_{X}}\left(x-\mu_{X}\right)\right]}^{2}}{\sigma_{Y}^{2}\left(1-r^{2}\right)}\right)$$

This is a normal density with mean $\mu_{Y}+r\left(x-\mu_{X}\right)\sigma_{Y}/\sigma_{X}\;$ and variance $\sigma_{Y}^{2}\left(1-r^{2}\right)$. The former is the conditional mean, and the latter is the conditional variance of Y given X. The conditional mean is a linear function of X. The conditional variance does not concern the baseline variance $\sigma_{X}^{2}$, and it decreases as |r| increases.

We note that if the joint distribution of (X,Y) is bivariate normal, then the marginal distribution of X is normal, and the marginal distribution of Y is also normal. However, the opposite is not true. That is, even if both X and Y are both normal, the joint distribution of (X,Y) may not be bivariate normal.

In practice, this means that if we observe either X or Y as being not normally distributed, then the joint distribution of (X,Y) is not bivariate normal, and therefore the conditional variance $\sigma_{Y}^{2}\left(1-r^{2}\right)$ does not apply. Even if we observe that both X and Y are normal, we still cannot ascertain that the joint distribution of (X,Y) is bivariate normal, hence the conditional variance $\sigma_{Y}^{2}\left(1-r^{2}\right)$ still may not apply.

In the above, we have implicitly assumed no interaction between baseline score and treatment group. In practice, this is another assumption to be verified. If there is such an interaction, r will be different in both arms.

## Appendix A Supplementary data

Data Profile Data Profile
